# Two cases and a review of *Streptococcus pyogenes* endocarditis in children

**DOI:** 10.1186/1471-2431-14-227

**Published:** 2014-09-10

**Authors:** Danielle R Weidman, Hilal Al-Hashami, Shaun K Morris

**Affiliations:** Department of Pediatrics, The Hospital for Sick Children, 555 University Avenue, M5G 1X8 Toronto, Ontario Canada; Division of Infectious Diseases, The Hospital for Sick Children, Toronto, Canada; University of Toronto, Toronto, Canada; Peter Gilligan Research Institute, Toronto, Canada

**Keywords:** Endocarditis, *Streptococcus pyogenes*, Group A *Streptococcus*, Toxic shock

## Abstract

**Background:**

Infective endocarditis is a rare diagnosis in pediatrics. Group A beta-hemolytic *Streptococcus pyogenes* is known to cause a range of type and severity of infections in childhood. However, *S. pyogenes* is a rarely described cause of endocarditis in children. This paper presents two cases of *S. pyogenes* endocarditis and the largest and most up-to-date review of cases previously reported in the literature.

**Case presentation:**

Here we describe two pediatric cases of *S. pyogenes* endocarditis with associated toxic shock. Case 1 was a previously well Caucasian 6-year-old female who presented with sepsis. Case 2 was an 8-month-old South Asian female who presented with sepsis and pneumonia. We present a review of the literature since the beginning of the antibiotic era of this unusual cause of bacterial endocarditis in children.

**Conclusion:**

In addition to the two cases presented here, a total of 13 children have been reported since 1940 with endocarditis caused by *S. pyogenes* for which clinical details are available. Although few cases exist, literature review reveals a high mortality rate (27%) and the majority of patients who recovered had residual morbidities. We emphasize the importance of considering a diagnosis of endocarditis in cases of *S. pyogenes* sepsis or toxic shock in order to ensure early diagnosis and timely treatment, which are necessary for good outcomes. This information is relevant to both general and subspecialty pediatricians, especially emergency room and infectious disease physicians.

## Background

Infective endocarditis (IE) is a rare diagnosis in children. Group A beta-hemolytic *Streptococcus pyogenes* can cause a range of type and severity of infections in childhood including invasive, toxin-mediated, and immune-mediated disease. However, *S. pyogenes* is a rarely described cause of IE in children. The estimated proportion of IE caused by *S. pyogenes* in children under age 21 is less than 3% [1]. In addition to the two cases presented here, a total of 13 children have been reported since 1940 with endocarditis caused by *S. pyogenes* for which clinical details are available. Here we describe two cases of IE caused by *S. pyogenes* in children with associated toxin-mediated disease, and present a review of the literature since the beginning of the antibiotic era.

## Case presentation

### Case 1

A 6-year-old previously healthy unvaccinated Caucasian female was seen at a rural emergency department (ED) with a 3-week history of fevers and a 3-day history of lethargy, poor oral intake, and erythematous rash. Following discharge from the ED, she re-presented two days later to the same ED with fever and signs consistent with sepsis. She was transferred to the intensive care unit (ICU) of our tertiary care center after fluid rehydration and one dose of ceftriaxone. At least 3 of her 6 siblings had a recent history of pharyngitis, although only one was seen by a physician, diagnosed clinically as strep throat, and treated. On examination she had a systolic murmur, painful purple nodules of the fingers and toes, and erythema of the palms and soles. She was hypotensive and required dopamine and epinephrine infusions to maintain blood pressure. White blood cell count was 18.7×10^9^/L with 73% neutrophils. Initial electrocardiogram (ECG) was normal. Antimicrobial therapy initiated on admission was vancomycin and meropenem. Transthoracic echocardiogram (echo) was done due to clinical stigmata suspicious for IE, which demonstrated a mobile mass on the posterior mitral leaflet (0.7 cm × 0.5 cm) and a small atrial septal defect (ASD)/patent foramen ovale (PFO). Head computed tomography (CT) revealed multiple bilateral asymmetric hypoattenuating foci, presumed to be septic emboli or watershed infarcts. Once the diagnosis of IE was made, gentamicin was added. Three hours after admission to the ICU, cardiac monitor abnormalities prompted a repeat ECG, which showed accelerated junctional rhythm and atrioventricular (AV) dissociation. The decision was made not to operate, as her echo showed good function and no aortic root abscess. On day 2 of admission, blood cultures drawn at the community hospital came back positive for *S. pyogenes*. Penicillin (400,000 units/kg/day) and clindamycin were started; gentamicin was discontinued. By day 3 of admission, she had clinically improved and vancomycin and meropenem were stopped. Blood cultures drawn on admission to our institution were sterile. She improved rapidly on penicillin and by day 5 of admission, the suspected toxin-mediated process had resolved, and clindamycin was discontinued. Liver and renal function remained normal. She was discharged home 8 days after presentation and completed 6 weeks of intravenous penicillin (400,000 units/kg/day). Complete resolution of the vegetation was seen on 3-month follow-up echo.

### Case 2

A previously well 8-month-old South Asian female presented to a community hospital with a clinical picture suggestive of sepsis. She had a 1-week history of fevers and viral respiratory prodrome and a 2-day history of lethargy and poor oral intake. On initial presentation, she had increased work of breathing and chest x-ray demonstrated a right pleural effusion. She was initially treated with vancomycin, cefotaxime, and oseltamivir. On day 2 of hospitalization, she was intubated due to worsening lethargy, poor perfusion, and acidosis. She was then transferred to the ICU at our tertiary care facility where hemodynamic support was provided and a chest tube was placed. Blood cultures drawn from the community hospital grew *S. pyogenes* and penicillin (300,000 units/kg/day) and clindamycin were started. Repeat blood cultures at our institution were also positive for *S. pyogenes*. Due to hemodynamic instability, a transthoracic echocardiogram was performed to assess cardiac function. The study was limited by clinical instability but did not show evidence of endocarditis. In addition to hypotension, she developed multi-organ dysfunction including acute renal failure, elevated liver enzymes, and thrombocytopenia. On day 6 of illness, she had left hand twitching and bilateral abnormal eye movements. Head CT showed extensive bilateral asymmetric foci of white matter diffusion restriction consistent with watershed infarcts and right parietal lobe findings suggestive of hemorrhagic septic emboli. Repeat transthoracic echocardiogram showed a tricuspid valve vegetation and a PFO. High dose penicillin was continued and blood cultures became negative after one week of antibiotic therapy. She was transferred to the ward on day 18 of illness and discharged home after 34 days total. Penicillin (300,000 units/kg/day) was continued to complete 6 weeks, and she had complete resolution of symptoms on follow-up.

### Discussion

Infective endocarditis is unusual in children and carries significant morbidity and mortality. *S. pyogenes* is a rare cause of this condition. This organism is a virulent cause of endocarditis, as the infection has the potential to progress despite appropriate therapy, which can ultimately lead to death [2].

Prior to the antiobiotic era, infective endocarditis was nearly always fatal, regardless of the causal pathogen [3]. Incidence of endocarditis caused by *S. pyogenes* has declined significantly since the introduction of antiobiotics, largely due to antibiotic treatment of local pyogenic infections preventing bacteremia and seeding of the endocardium [4].

While underlying congenital heart disease is considered a major risk factor for endocarditis [3], the cases we present and the majority of those with *S. pyogenes* endocarditis reported in the literature occurred in children with structurally normal hearts. This is consistent with the epidemiology of other causes of endocarditis in children, which has shifted in recent years to affect a higher proportion of children without underlying structural heart disease [1].

Bacteremia caused by *S. pyogenes* comes from a primary source, which may be cellulitis, pharyngitis, endometritis, pneumonia, or other localized pyogenic infection. In children, the primary source is most commonly pharyngitis or skin lesions [5]. Superinfection of varicella skin lesions with *S. pyogenes* is the most common focus of infection leading to endocarditis by this organism [6, 7]. However, endocarditis as a complication of varicella is very unusual [8]. In Case 1, the portal of entry was likely pharyngitis. Although less clear in Case 2, the initial infection may have been pneumonia.

*S. pyogenes* can produce multiple exotoxins, which have potential to cause end organ damage or release cytokines that can cause tissue injury [5]. The syndrome of toxic shock caused by *S. pyogenes* endocarditis is uncommon, especially in children. Both cases we describe are consistent with clinical features of toxic shock. Case 2 fits the accepted definition of toxic shock [9], as she had hypotension as well as multi-organ involvement including renal impairment, liver dysfunction, coagulopathy, and respiratory distress. Case 1 had features of toxic shock including hypotension and a generalized rash; however, the clinical picture does not meet formal criteria [9]. There are few cases of toxic shock associated with *S. pyogenes* endocarditis in the literature. The case reported by Liu et al. required intubation, vasopressors, and fluid resuscitation, although toxic shock is not specifically discussed [7]. As well, Winterbotham et al. describe a case with hypotension and respiratory failure; however, the details are unclear [5]. In general, few cases described previously were as clinically unstable as those presented here.

Since these two cases presented to our tertiary care facility within two weeks of each other, we analyzed the strains of *S. pyogenes* to see if the two cases may have been caused by identical strains. The technique used was emm typing by PCR done by the National Microbiology Laboratory [10, 11]. The strains were shown to be different.

### Review of the literature

We conducted an electronic literature search using PubMed and restricted to English language but did not place restrictions on search dates. We further examined reference lists in retrieved articles for additional citations not found by the electronic search.

In addition to the two cases presented above, a total of 13 children have been reported since 1940 with endocarditis caused by *S. pyogenes* for which clinical details are available. In this review, cases from the antibiotic era were included (Table 1). As such, this resulted in the exclusion of several cases that did not have clinical information available, or were presented with inconsistencies or ambiguous nomenclature (Table 1).

**Table 1:** **Cases of** ***S. pyogenes*** **endocarditis from 1940 to present for which clinical features are available**

Case ^a^ # [ref]	Year	Age, Sex	Pre-existing cardiac abnormalities	Preceding infection	Valve(s) affected	Complications	Emboli	Surgery	Outcome
1 [12]	1942	2y, M	None	None	Mitral	• Splenomegaly	Brain, Peripheral, Renal, Spleen	No	Death
						• Bronchopneumonia			
						• Painful swollen joints			
2 [3]	1975	6 m, M	None	Meningitis	Aortic	• Microscopic hematuria	None	No	Recovered with cardiomegaly and decreased cardiac function
						• LVH^b^			
						• Aortic insufficiency and CHF^c^			
						• Left sinus of Valsalva aneurysum			
3 [13]	1977	14y, M	VSD^d^ closed surgically at age 6, aortic stenosis	Tonsillitis	Aortic	• Myocardial abscesses	Not reported	No	Death
						• Rupture of sinus of Valsalva into left ventricle			
4 [4]	1980	16y, M	None	Febrile sore throat	Aortic	• Respiratory distress requiring intubation and ventilation	Peripheral, Renal	No	Recovered
						• Renal failure (resolved)			
5 [2]	1984	2y, M	Pulmonic stenosis	None	Aortic and Mitral	• Pulmonary edema	Brain, Spleen	No	Death
						• LVH^b^			
						• Aortic insufficiency			
						• Proliferative glomerulonephritis			
6 [6]	1988	4 m, F	None	Varicella	Aortic	• Para-aortic abscess	None	No	Death
						• CHF^c^ and pulmonary edema			
7 [14]	1988	4y, F	None	None	Aortic	• Polyarthritis	None	Yes	Recovered with small VSD^d^
						• Aortic regurgitation			
						• Sinus of Valsalva aneurysm			
8 [7]	1992	3y, F	None	None	Mitral	• Left-sided hemiparesis	Brain, Peripheral	No	Recovered with left-sided weakness and mitral regurgitation
						• Microscopic hematuria			
						• Mitral regurgitation			
						• Pericardial effusion			
						• Required vasopressors and intubation			
9 [5, 15]	1998	14y, M	None	None	Mitral	• Right cerebral infarction	Brain	No	Recovered with left-sided weakness
10 [5]	1999	5 m, M	None	Varicella	Mitral	• Seizure	Peripheral	Yes	Recovered
						• Respiratory failure			
						• Hypotension			
						• Mitral regurgitation with anterior leaflet tear			
						• Aortic root abscess			
						• Toe autoamputation			
11 [16]	2000	4y, M	None	None	Mitral	• Mitral regurgitation	Left foot	Yes	Recovered
						• Necrosis of 2 toes requiring amputation			
12 [16]	2000	2y, M	None	Varicella	Aortic	• Aortic root abscess	Brain, Peripheral	Yes	Recovered with left hemiplegia
						• Aortic valve perforation			
						• Focal seizures			
13 [8]	2000	3y, M	None	Varicella	Aortic	• Joint pain	Peripheral	Yes	Recovered
						• Bilateral pulmonary infiltrates			
						• Elevated LFTs^e^			
						• Aortic insufficiency			
						• Left ventricular dilatation leading to CHF^c^			

^a^Includes all cases from which clinical features, demographics, and outcome can be extracted. There exists an additional group of cases of possible *S. pyogenes* endocarditis in children for which sufficient clinical data is not available [1, 17–21]. As well, a few cases were excluded due to ambiguity [21, 22].

^b^LVH = left ventricular hypertrophy.

^c^CHF = congestive heart failure.

^d^VSD = ventricular septal defect.

^e^LFTs = liver function tests.

Out of 15 cases of *S. pyogenes* endocarditis with clinical information available (including those presented above), 10 were male (67%) and 5 were female (33%). Ages range from 4 months to 16 years, with a median age of 3 years. Only 2 patients had known pre-existing heart defects. Varicella was a preceding infection in 4 cases (27%), and 6 patients (40%) had no known preceding infection. The most common valves involved were mitral and aortic, with each accounting for approximately 50%. Case 2 is the only reported case in this population with IE affecting the tricuspid valve. Embolic phenomenon occurred in 11 patients (79%), with one not documented. Five patients had cardiac surgery (33%). The mortality rate was 27% and the majority of patients who recovered had residual morbidities.

## Conclusions

In summary, we present a rare combination of endocarditis caused by *S. pyogenes* in two children with previously normal hearts admitted to the same hospital in a two-week time period, both with signs of toxic shock. Our cases show that in cases of *S. pyogenes* sepsis or toxic shock, consideration should be given to a concurrent diagnosis of endocarditis, even if the heart is structurally normal. When treated with appropriate antimicrobial therapy and supportive care in a timely manner, even severe presentations of *S. pyogenes* endocarditis and toxic shock can have excellent outcomes.

## Consent

Written informed consent was obtained from the patients’ parents for publication of these case reports. Consent was documented in the patients’ charts according to The Hospital for Sick Children’s Consent for Publication/Presentation of Case Reports Policy. Copies are available for review upon request.

## Abbreviations

ASD: Atrial septal defect
AV: Atrioventricular
CT: Computed tomography
ECG: Electrocardiogram
Echo: Echocardiogram
ED: Emergency department
ICU: Intensive care unit
IE: Infective endocarditis
PFO: Patent foramen ovale.
